# Broad cognitive complaints but subtle objective working memory impairment in fibromyalgia patients

**DOI:** 10.7717/peerj.5907

**Published:** 2018-11-21

**Authors:** Marina Pidal-Miranda, Alberto Jacobo González-Villar, María Teresa Carrillo-de-la-Peña, Elena Andrade, Dolores Rodríguez-Salgado

**Affiliations:** 1 Department of Clinical Psychology and Psychobiology, Universidad de Santiago de Compostela, Santiago de Compostela, Spain; 2 Department of Social, Basic and Methodological Psychology, Universidad de Santiago de Compostela, Santiago de Compostela, Spain

**Keywords:** Chronic pain, Dyscognition, Working memory, Cognitive complaints, Fibromyalgia, Fibrofog, Cognition, Neuropsychological performance, Cognitive dysfunction

## Abstract

**Background:**

Cognitive dysfunction in fibromyalgia (FM) encompasses objective cognitive difficulties, as measured in neuropsychological tests, and self-reported cognitive complaints. Although it has been suggested that FM patients display problems in working memory, the data are inconsistent, and the overall working memory status of the patients is unclear. It is also not clear whether the working memory problems are related to cognitive complaints or how the dyscognition is affected by the characteristic clinical symptoms of FM.

**Methods:**

To clarify these aspects, we explored the neuropsychological performance for different components of working memory and the subjective self-perception of cognitive status in a sample of 38 women with FM. They were compared with a matched group of 32 healthy women.

**Results:**

Our findings suggested that the FM patients do not differ from healthy controls in their overall working memory functioning. Only a poor performance was found in a single task of visuospatial working memory, mediated by the presence of depressive symptoms, fatigue and pain. The FM patients also displayed a higher level of perception of cognitive difficulties than healthy controls, and this difference was mediated by depression and fatigue. Furthermore, cognitive complaints in FM patients were only associated with a lower verbal WM capacity.

**Discussion:**

FM patients have a subtle specific impairment in their working memory functioning, as well as elevated concern about their cognitive status. These findings suggest a disconnection between neuropsychological performance and subjective complaints. In FM patients, clinical variables such as pain, fatigue, and depression play an important role in dyscognition, as assessed by both objective and subjective measures, and should be taken into account in future research.

## Introduction

Fibromyalgia (FM) is a chronic disease of uncertain aetiology that affects 2.7% of the population, with a female-to-male ratio of 3:1 (Queiroz, 2013). It is characterized by widespread musculoskeletal pain and other symptoms such as fatigue, stiffness, mood disturbance, restless sleep and cognitive dysfunction (Crombez et al., 2013). Cognitive dysfunction in FM, also called dyscognition, encompasses objective cognitive difficulties observed in neuropsychological tests and also self-reported cognitive complaints (Glass, 2009).

Regarding objective cognitive difficulties, it has been proposed that patients with FM have particular problems in working memory (WM) because they perform poorly in tasks involving distraction (Katz et al., 2004) or complex and rapidly changing environments (Glass, 2009; Ceko, Bushnell & Gracely, 2012). However, according to multicomponent models, WM represents a multifaceted construct that requires full exploration. One of the most influential models (Baddeley & Hitch, 1974; Baddeley et al., 1986; Baddeley, 1996; Miyake & Shah, 1999) proposes that WM is composed of a central executive and two subsystems for temporary storage and rehearsal of auditory-verbal and visuo-spatial information, the phonological loop and the visuo-spatial sketchpad, respectively. The central executive system converges aspects of attention, memory and executive functions, and is also assumed to be fractionable in different components, including the capacity to maintain, monitor, manipulate and update information, inhibition, and attentional flexibility.

Several studies have revealed that FM patients show deficits in the phonological loop component of WM (Roldán-Tapia et al., 2007; Leavitt & Katz, 2008; Munguía-Izquierdo et al., 2008; Di Tella et al., 2015), in the visuospatial sketchpad (Luerding et al., 2008), and in different aspects of the central executive system related to WM, such as the capacity to update and inhibit information (Seo et al., 2012; Akdoğan et al., 2013; Cuevas-Toro et al., 2014; Martinsen et al., 2014; Cherry et al., 2014; Coppieters et al., 2015; Tesio et al., 2015; Di Tella et al., 2015). Other authors have either not found any evidence of altered performance for these WM components in FM patients (Landrø, Stiles & Sletvold, 1997; Suhr, 2003; Walteros et al., 2011; Glass et al., 2011; Ceko, Bushnell & Gracely, 2012; Mohs et al., 2012; Kim et al., 2012; Veldhuijzen, Sondaal & Oosterman, 2012; Schmidt-Wilcke et al., 2014; Tesio et al., 2015), or have explained it by depression or other symptoms of FM such as fatigue and pain (Landrø, Stiles & Sletvold, 1997; Dick, Eccleston & Crombez, 2002; Suhr, 2003; Munguía-Izquierdo et al., 2008; Kim et al., 2012; Gelonch et al., 2016). Thus, it is difficult to reach conclusions about the presence of WM deficits in FM patients. Furthermore, the variety of tasks used to assess WM and the diversity of samples in previous research (Berryman et al., 2013) hinder demonstration of the overall functioning of FM patients in WM. To the best of our knowledge, no research to date has evaluated the different components of WM together in the same group of FM patients, as evidenced in recent reviews and meta-analyses (Gelonch et al., 2013; Berryman et al., 2013; Wu et al., 2018).

Regarding subjective cognitive complaints, the term fibrofog is used to refer to the loss of mental clarity and to the impaired attention and memory, which are frequently reported by FM patients. It is one of the most prevalent symptoms of FM and is often considered more disabling than the pain itself. Despite being a very common and disruptive symptom, cognitive complaints are not well studied and seem to have been overlooked in research on FM dyscognition (Glass, 2009). However, clinicians and researchers highlight the need for further research into the cognitive dysfunction experienced by FM patients, and this symptom has in fact been incorporated into the latest diagnostic criteria of FM (Wolfe et al., 2010).

Previous research has suggested that characteristic symptoms of the disease, such as depression, fatigue and pain have an impact on subjective cognitive complaints in FM patients (Suhr, 2003; Castel et al., 2008; Williams, Clauw & Glass, 2011; Kravitz & Katz, 2015; Gelonch et al., 2016; Gelonch et al., 2017). This influence could be explained by the fact that the affective and physical variables of the disease may exacerbate the amount of perceived effort required to perform a cognitive task (Bar-On Kalfon et al., 2016). Thus, the subjective cognitive perception of the patients would be altered, increasing their complaints, but not necessarily their objective performance. This would contribute to a disconnection between self-reported cognitive complaints in FM patients and measurable objective deficits.

Few studies to date have investigated the extent to which cognitive complaints can reliably indicate impaired cognitive function in FM; the findings of these and of studies with other clinical populations (Alegret et al., 2015), older adults (Crumley, Stetler & Horhota, 2014; Chin et al., 2014; Burmester, Leathem & Merrick, 2016) and working middle-aged adults (Stenfors et al., 2013) have been controversial. Some studies have observed a relationship between FM patients’ complaints and the performance of neuropsychological tests (Park et al., 2001; Tesio et al., 2015), supporting the use of complaints as a valid indicator of cognitive problems. By contrast, other authors have highlighted the disconnection between the subjective experience of cognitive problems and the objective reality of cognitive performance in FM patients (Walitt et al., 2016; Gelonch et al., 2016).

In summary, the overall working memory status of FM patients is not clear, as the different components of working memory have not yet been evaluated in the same group of patients. In addition, the role of clinical symptoms of FM on dyscognition has not yet been determined. It is also not clear whether cognitive complaints in FM patients are related to working memory performance in neuropsychological tests. Therefore, the aims of this study were (1) to determine whether there are significant differences between FM patients and healthy control subjects in a series of objective WM measures and subjective cognitive measures, analyzing the effect of pain, depression and fatigue on the possible differences; (2) to explore the relationship between the objective WM and subjective cognitive measures in FM patients.

## Materials and Methods

### Participants

Thirty-eight women diagnosed with fibromyalgia and 33 healthy controls (HC), women matched for age, education, laterality and menopausal status, were enrolled. The participants gave their written informed consent for their involvement in the study, approved by the Galician Autonomous Committee for Research Ethics (2013/582), and conducted in accordance with the Declaration of Helsinki. Patients were referred from different medical centres and FM associations in Galicia (NW Spain). Healthy controls were recruited from the community. The participants were aged between 28 and 64 years old and received 25 € to cover travelling expenses. The inclusion criterion for the FM group was a diagnosis of the disease by a rheumatologist, meeting the diagnostic criteria established in 1990 (Wolfe et al., 1990). A history of any medical condition associated with cognitive dysfunction, mental illness or psychiatric disorders (except for anxiety and depression symptomatology) was an exclusion criterion for all participants. In addition, healthy controls (HC) should not have any chronic pain condition. For ethical reasons, patients were not asked to withdraw prescribed medical treatments. Demographic and clinical characteristics of the groups are shown in Table 1.

**Table 1:** Characteristics of FM patients and controls.

	FM patients healthy controls		*t*, *χ*^2^ or *U*	*p*
	*N* = 38	*N* = 33		
Age *M (SD)*	47.71 (9.63)	47 (9.01)	*U* = 609	.835
Education (%)			*X*^2^ = 0.098	.952
Primary school	36.8	33.3		
High school	36.8	39.4		
Higher studies	26.3	27.3		
Menopausal women (%)	47.4	45.5	*X*^2^ = 0.026	.872
Right handed (%)	97.4	97	*X*^2^ = 2.02	.364
BDI *M (SD)*	24.11 (13.46)	10.08 (5.54)	*t* = − 5.56	**<.001**
VAS (cm)				
Pain *M (SD)*	6.68 (1.71)	2.98 (3.25)	*t* = − 6.048	**<.001**
Fatigue *M (SD)*	7.70 (1.99)	3.22 (2.55)	*t* = − 7.36	**<.001**
5DT Reading (*s*) *M (SD)*	22.65 (4.63)	20.79 (3.88)		
n-back RT (*s*)				
1-back *M (SD)*	0.60 (0.12)	0.55 (0.12)		
2-back *M (SD)*	0.64 (0.12)	0.59 (0.13)		
Medication				
Non-opioid analgesics	23	2		
Pregabalin/Gabapentin	6	1		
Antidepressants	19	3		
Opioids	11	0		
Anxiolytics	15	5		

**Notes.**

M (SD) mean (standard deviation) BDI Beck Depression Inventory VAS Visual Analogue Scale 5DT 5-Digit Test RT Reaction Time

### Measures

#### Sociodemogaphic and clinical information

Clinical and sociodemographic data on the participants were obtained via a semi-structured interview. The intensity of pain and of fatigue experienced in the previous week were measured on 0–10 *Visual Analogue Scales* (VAS). Depressive symptoms were assessed using the Spanish version of the *Beck Depression Inventory* (Beck, Steer & Brown, 1996). The handedness of participants was assessed by administration of the *Edinburgh Handedness Inventory* (Oldfield, 1971).

#### Working memory assessment

A multicomponent approach was used to assess WM (Baddeley et al., 1986; Miyake & Shah, 1999; Tirapu-Ustárroz et al., 2005; Verdejo-García & Bechara, 2010). The visuospatial sketchpad, the phonological loop and different aspects of the central executive system related to WM (such as the capacity to maintain, monitor, manipulate and update information, as well as inhibition and attentional flexibility) were assessed by the tasks described below.

The *Digit self-Ordering Task* (DOT; Sunderland, Harris & Gleave, 1984; Petrides et al., 1993) allowed us to obtain information about the monitoring process that the subject required for successful completion of tasks. Participants were asked to say out loud numbers between 1 and 10 at random, without repeating or forgetting any digits in ten trials. The sum of omission errors and repetitions over the ten trials was recorded as the final score.

The *5-Digit Test* (Sedó, 2004) was used to assess aspects of WM related to the executive system. The test is described as a numerical Stroop task, with the advantage of displaying minimal verbal content and thus permitting its application in multilingual contexts, as in the case of the region where the present study was carried out. Further, it assesses both the ability to cope with interference and to alternate between mental processes—flexibility-, and also the speed of cognitive processing. It is composed of four subtests: in part 1 (*Reading*) participants were asked to read the digit presented in a series of text boxes, each containing as many repetitions of the digit as it indicates itself. In part 2 (*Counting*), the boxes contained asterisks and the participants were asked to state the number of these in each box. In part 3 (*Focusing*), the boxes were similar to those in part 1, except the identity of the digit in each box did not correspond to the number of digits in the box. Participants were then asked to state the number of digits and to ignore their identity. In part 4 (*Switching*), an extra clue indicates whether the participant must report the number of digits or their identity (reading or counting). In all parts of the test, performance was measured in terms of the time required to complete the task. *Inhibition* (*Focusing* minus *Reading*) and *Flexibility* (*Switching* minus *Reading*) scores were calculated to measure WM components related to the executive system.

The *Letter-Number Sequencing Subtest* of the Spanish version of the *Wechsler Memory Scale III* (Wechsler, 1997) was used to provide data on the maintenance and manipulation of verbal information capacity in WM. A series of mixed and randomly ordered lists of letters and numbers of increasing length were presented orally. The participants were asked to repeat the lists in a certain order: first stating the numbers in ascending order followed by the letters in alphabetical order. The span score was recorded.

The *Spatial Localization Subtest* of the Spanish version of the *Wechsler Memory Scale III* (Wechsler, 1997) was used to assess aspects of the visuospatial sketchpad and the capacity to manipulate visuospatial information while in temporary storage. This task consists of nine cubes placed on a board that the examiner taps in a specific order. The participants were asked to observe the sequence of blocks tapped and repeat it in the *Forward* part of the task. In the *Backwards* task, the subjects were asked to repeat the sequence in reverse order. The task started with a series of two blocks and gradually increased in length. *Forward* and *Backwards* span scores were recorded.

The *Digit Subtest* of the Spanish version of the *Wechsler Memory Scale III* (Wechsler, 1997) was administered to evaluate the phonological loop and the ability of subjects to manipulate verbal information while in temporary storage. Participants were asked to repeat chains of digits of increasing length in the given order in the *Forward* task and in reverse order in the *Backwards* task. *Forward* and *Backwards* span scores were recorded.

**Figure 1:**
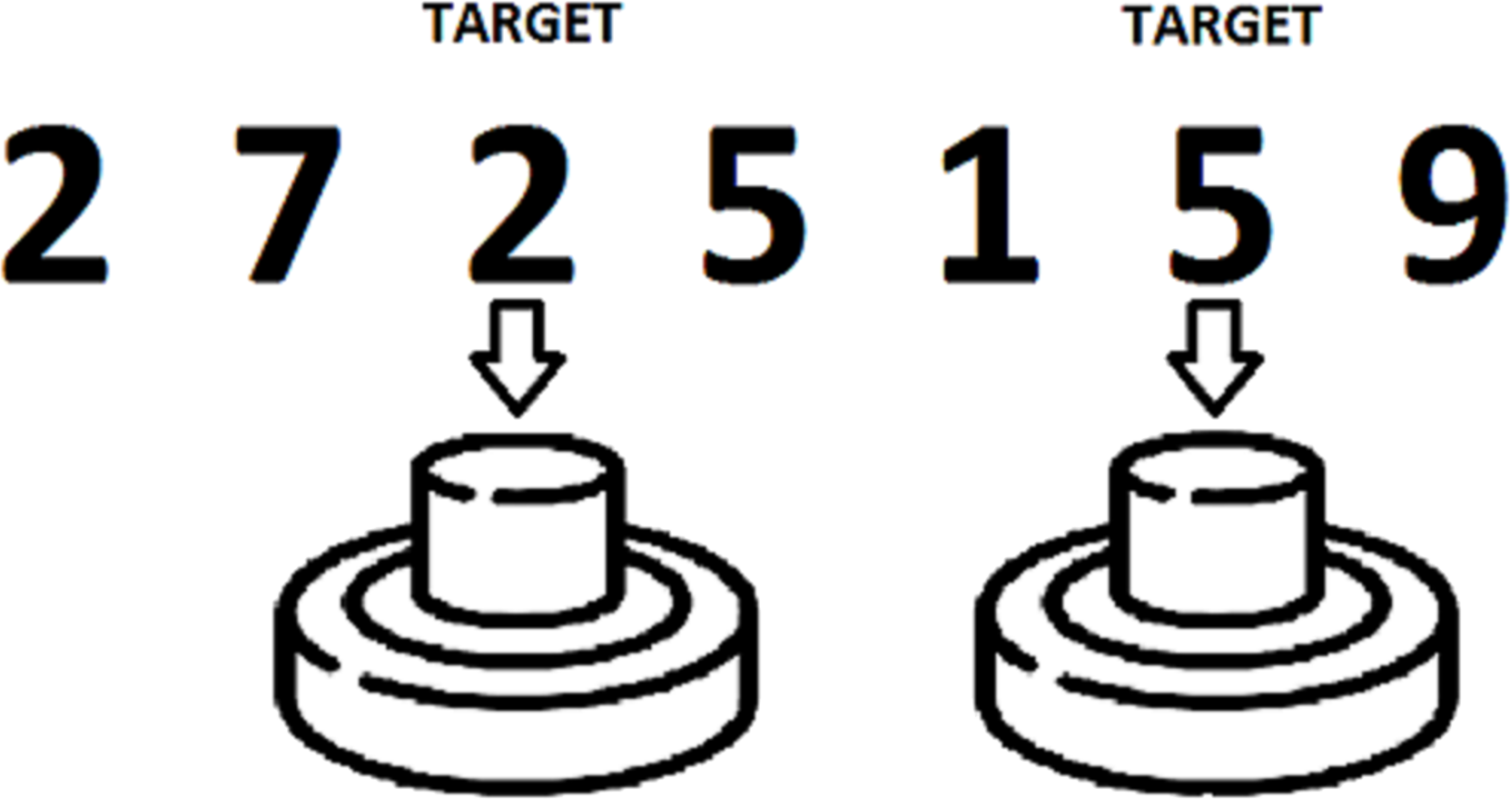
Example of a 2-back task.

Visual *1-back* and *2-back* tasks were also administered, in order to assess the ability to maintain, monitor, manipulate and update information in WM. PsychoPy software (Peirce, 2008) was used to design and present the tasks on a computer. Subjects were required to monitor a sequence of digits (0 to 9) presented one by one, and to press a button in a response box with the index finger of their dominant hand when a target stimulus appeared. The target stimuli were the same numbers presented one trial (*1-back* condition) or two trials (*2-back* condition) before (Fig. 1). Each task consisted of 220 trials, with a 30% of target stimuli. The percentage of correct answers and the number of false positives in each task were recorded for posterior analysis.

In addition to the WM measures described above, the processing speed was also measured using the *5-Digit Test Reading* subtest score and reaction times in the *1-back* and *2-back* tasks. Although processing speed is not a subcomponent of working memory, it is present in many WM tasks. To ensure that our results reflected the performance of the participants strictly in WM components, we controlled for possible between-groups differences in processing speed.

#### Subjective cognitive complaints

With a view to obtaining information about the overall cognitive complaints, the participants were questioned about “Trouble thinking or remembering” in item I.2 of the *Fibromyalgia Survey Questionnaire* (Carrillo-de-la Peña et al., 2015). The answer was coded as 0 (not present), 1 (slight problems), 2 (moderate problems) and 3 (severe problems).

As subjective reports of cognitive impairment are considered to be accurate if the subjective questionnaire evaluates specific types of behaviour (Hertzog et al., 2000), the Spanish Version of the *Memory Failures of Everyday Questionnaire* was also administered (Sunderland, Harris & Gleave, 1984; Lozoya-Delgado, Ruiz-Sánchez de León & Pedrero-Pérez, 2012). The *MFE-30* is a Likert 5-point (between ‘never’ and ‘very often’) questionnaire, comprising 30 items related to complaints in different cognitive domains. The *MFE-30* Total score (range 0–120) was calculated as the sum of all items. This score was ranked into four categories: 0–7, 8–35, 36–50 and over 50, indicating optimal performance, normal, mild deterioration and moderate deterioration respectively. In order to obtain additional information from the *MFE-30* questionnaire, two additional scores were considered: one was calculated as the sum of items related to general functioning (General Function score), and the other was determined as the sum of items linked to more specific activities in daily living (Daily Life score) (Lozoya-Delgado, Ruiz-Sánchez de León & Pedrero-Pérez, 2012).

### Procedure

A psychologist trained in neuropsychological assessment collected all the data, following a standardized protocol. Demographic and clinical information were obtained during the evaluation session, after participants had provided written informed consent to take part in the study. Information about laterality and subjective complaints was obtained before participants performed the paper and pencil WM tests and the computerized *n-back* tasks. The order of administration of the tasks was maintained for all the participants.

Data collection sessions took place in the Faculty of Psychology of the University of Santiago de Compostela. Each session lasted approximately 50 min, depending on the ability of each participant.

### Statistical analysis

Means and standard deviations were used to describe the quantitative variables, while absolute frequencies and percentages were used for the qualitative measures. Normality of variables was tested with the Shapiro–Wilk test. Differences between FM and HC groups in clinical and sociodemographic variables were analyzed using Student’s *t*-test or Chi-square test, depending on the type of variable considered. Group differences in processing speed were analyzed by multivariate analysis of variance (MANOVA) with *n-back* reaction times and *5-Digit Reading* subtest score as dependent variables.

Differences between the groups in the WM scores were tested for significance by MANOVA. Univariate analyses were also performed to study each measure independently. The size of the effect was measured using the eta-squared coefficient (*η*2), in which values of >0.01, >0.06 and >0.14 were respectively defined as small, medium and large. Analysis of covariance (ANCOVA) was used to statistically control the effect of depression, fatigue and pain when group differences in WM measures were found to be significant. Bonferroni correction for multiple comparisons was applied.

In order to examine the individual performance of FM patients in WM tasks that could be masked in group analyses, and to further investigate the clinical context of these patients, individual scores were also analyzed. The *Z* scores for fibromyalgia patients were computed on the basis of our own control data, and the percentage of patients who performed poorly in each WM test (*Z* scores ≤ − 1) was then determined.

Differences between groups in the percentages of general subjective cognitive complaints, as measured by the *FSQ* I.2 score and the *MFE-30* rated Total score, were analyzed by Chi-square tests. On the other hand, differences between groups in detailed cognitive complaints, measured by the *MFE-30* Total, General Function, and Daily Life scores, were analyzed by Student’s *t*-test for independent samples. The effect size was calculated using Cohen’s *d*, for which values of <0.20, <0.50 and <0.80 were considered small, medium and large. Analysis of covariance (ANCOVA) was used to statistically control the effect of depression, fatigue and pain when group differences in cognitive complaints were significant.

Associations between WM performance and cognitive complaints for FM patients were quantified using Spearman’s bivariate correlations.

All statistical analyses were performed using *IBM SPSS Statistics 20* (IBM SPSS, 2011). Missing data were treated with the multiple imputation procedure implemented in SPSS. Differences in results were considered statistically significant at *p* <.05.

## Results

### Sample characteristics

There were no significant differences between the FM and HC groups in age, education, menopausal status or laterality. There were also no significant differences in processing speed measures, as indicated by the MANOVA with the *n-back* reaction times and the *5-Digit Reading* subtest scores [Wilks’ *λ* = 0.910 and *F*(1, 59) = 1.887, *p* = .142, *η*2 = 0.090]. However, as expected, FM patients obtained higher scores (*p* < .001) in measures of depression (BDI) and pain and fatigue (VAS). Detailed statistical data are shown in Table 1.

### Objective working memory performance

The overall MANOVA for the WM measures revealed no significant differences between the FM patients and the HC group [Wilks’ *λ* = 0.751 and *F*(1, 59) = 1.328, *p* = .234, *η*2 = .249]. Univariate tests showed that FM patients only scored significantly lower on the *Backwards Spatial Localization Subtest*, with a medium size effect (*F*(1, 59) = 5.474, *p* = .023, *η*2 = .085) (Table 2). This significant difference disappeared when the BDI [*F*(1, 58) = 0, *p* = .986, *η*2 = .0], VAS fatigue [*F*(1, 58) = .387, *p* = .536, *η*2 = .007] or VAS pain [*F*(1.66) = .242, *p* = .625, *η*2 = .004] scores were included as covariates.

**Table 2:** Working memory performance of the FM patients and healthy control groups.

	**FM Patients**	**Healthy Controls**	*p*	*η*^2^
	Mean (*SD)N* = 38	Mean (*SD)N* = 33		
DOT	8.18 (6.55)	8.48 (8.14)	.765	.002
5DT Inhibition	17.93 (6.43)	17.58 (6.68)	.558	.006
5DT Flexibility	30.65 (10.36)	30.95 (9.04)	.690	.003
L&N	5.03 (0.85)	4.85 (1.17)	.897	.000
Spatial L. Forward	5.55 (1.15)	5.42 (1.2)	.702	.002
Spatial L. Backwards	4.76 (1.28)	5.39 (1.29)	.023^*^	.085
Digit Forward	5.47 (1.006)	5.64 (1.14)	.655	.003
Digit Backwards	4.58 (1.004)	4.42 (0.969)	.405	.012
False Pos 1-back	1.78 (3.85)	0.82 (1.60)	.221	.025
False Pos 2-back	6.53 (9.04)	7.79 (10.36)	.613	.004
% Correct 1-back	92.97 (8.65)	96.09 (4.30)	.084	.050
% Correct 2-back	75.52 (10.62)	77.01 (12.03)	.609	.004

**Notes.**

DOT Digit self-Ordering Task 5DT 5-Digit Test L&N Letter-Number Sequencing Subtest Spatial L. Spatial Localization Subtest false Pos false positives

* *p* < .05.

**Figure 2:**
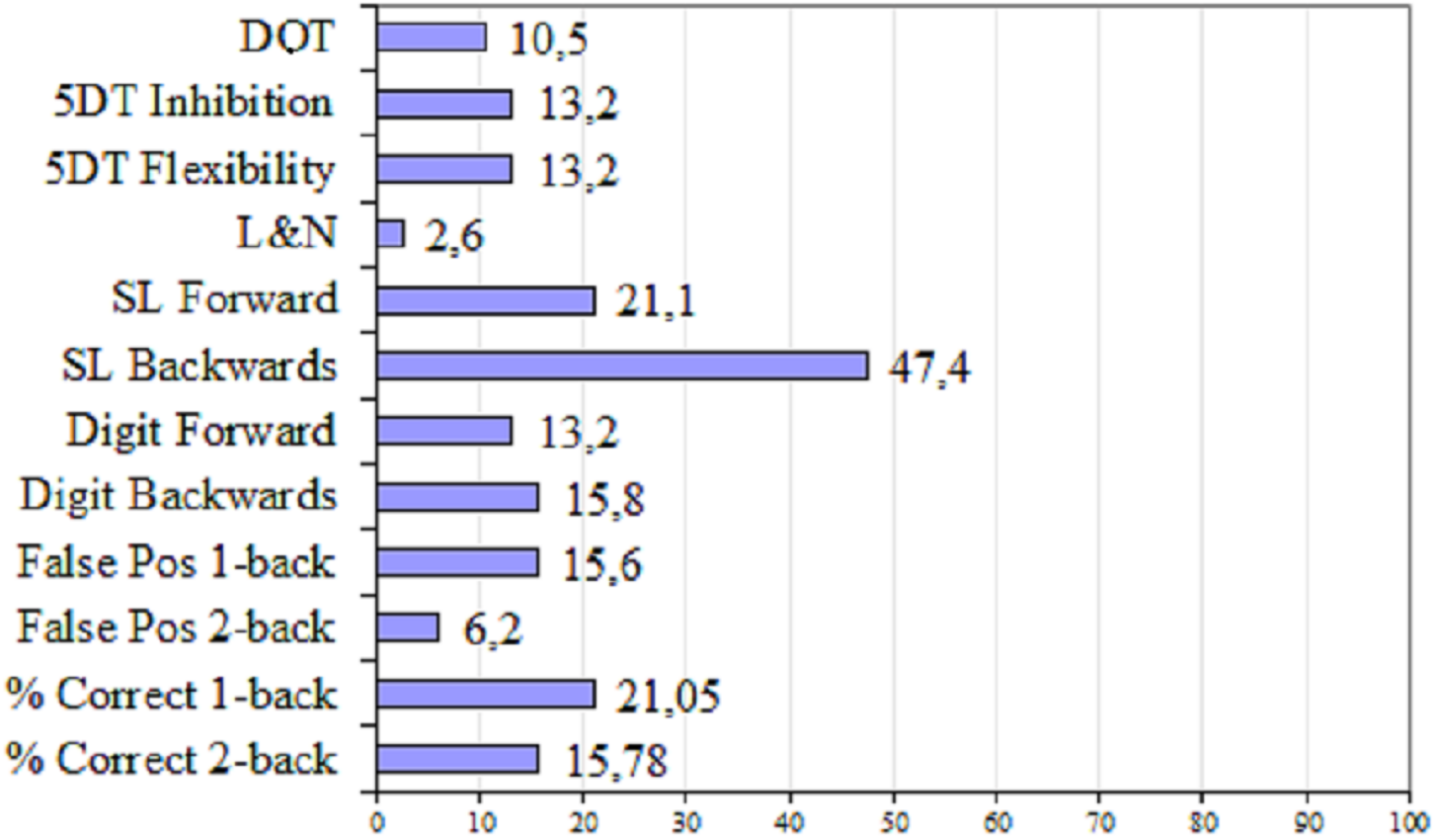
Percentage of FM patients with deficient performance in WM measures. DOT, *Digit self-Ordering Task*; 5DT, *5-Digit Test*; L&N, *Letter–Number Sequencing Subtest*; SL, *Spatial Localization Subtest*; False Pos, False positives

Regarding the analysis of individual WM performance in FM patients relative to healthy controls, Fig. 2 shows the percentage of FM patients with deficient performance (*Z* ≤  − 1) for each measure. The percentage of patients with deficient performance was highest (47%) for the span score obtained in the *Backwards Spatial Localization Subtest*. In addition, 21% of the patients exhibited poor performance in the *Forward Spatial Localization Subtest*, and in the correct responses of the *1-back* task.

### Subjective cognitive complaints

Compared to healthy controls, FM patients showed a higher percentage of cognitive complaints measured by the *FSQ I.2* item (*p* < .001): 78.9% of patients compared to 12.5% of HC reported moderate or severe cognitive problems. Furthermore, analysis of the *MFE-30* ranked Total score also revealed differences between groups (*p* < .001), with 71.1% of patients reporting mild or moderate impairment relative to 45.4% of healthy controls. All these data are shown in Table 3.

**Table 3:** Subjective cognitive complaints in FM patients and healthy controls.

	**FM Patients**	**Healthy Controls**	*t* or *X*^2^	*d*	*p*
	*N* = 38	*N* = 33			
**FSQ I.2 (%)**^a^			***X*^2^ = 33.01**		**<.001**
0: No problem	2.6	34.4			
1: Slight problems	18.4	53.1			
2: Moderate problems	50	12.5			
3: Severe problems	28.9	0			
**MFE-30 Scores**					
Rated total (%)			***X* = 22.66**		**<.001**
Optimal performance	2.6	3			
Normal performance	26.3	51.5			
Mild impairment	15.8	42.4			
Moderate impairment	55.3	3			
Total *Mean* (*SD*)	53.091 (25.62)	30.285 (12.90)	*t* = − 4.827	1.124	**<.001**
General *Mean* (*SD*)	34.118 (19.40)	17.293 (9.28)	*t* = − 4.755	1.106	**<.001**
Daily life *Mean* (*SD*)	18.973 (7.04)	12.992 (4.69)	*t* = − 4.256	0.999	**<.001**

**Notes.**

FSQ I.2 Fibromyalgia Survey Questionnaire “Trouble thinking or remembering” item MFE-30 Memory Failures in Everyday Questionnaire General general function

a One participant did not complete the FSQ.

Significant differences between groups were also found for cognitive complaints in the *MFE-30* Total score (*p* < .001), and in the General Function (*p* < .001) and Daily Life (*p* < .001) scores, with FM patients obtaining higher scores (Table 3). Results of ANCOVAs showed that the significant difference between groups in the *MFE-30* Total score disappeared when *BDI* [*F*(1, 53) = 2.819, *p* = .099] or fatigue VAS scores [*F*(1, 52) = 1.931, *p* = .171] were included as covariates, but not when pain VAS score was included [*F*(1, 54) = 8.187, *p* = .006]. Moreover, the additional measures of the *MFE-30*, General Function and Daily Life scores, followed the same pattern, with no significant differences when *BDI* [for the *MFE-30* General Function score: *F*(1, 53) = 2.017, *p* = .161; for Daily Life score: *F*(1, 53) = 3.727, *p* = .059] or fatigue scores [for General Function score: *F*(1, 52) = 1.541, *p* = .220; for Daily Life score: *F*(1, 52) = 2.287, *p* = .137] were added as covariates; however, the significant differences remained when the pain score was included [for General Function score: *F*(1, 54) = 8.701, *p* = .005; for Daily Life score: *F*(1, 54) = 4.567, *p* = .037].

### Relationship between objective working memory performance and subjective cognitive complaints

Correlations between the measures of objective WM performance and the subjective cognitive complaints evaluated by the *MFE-30* questionnaire were calculated for the FM patients (Table 4). The only significant (negative) correlations were found between the *Digit Forward* span score and the General Function, Daily Life and Total scores from the *MFE-30*.

**Table 4:** Correlations between WM performance and cognitive complaints in FM patients.

	MFE-30	MFE-30	MFE-30
	Total	General function	Daily life
DOT	−.211	−.164	−.316
5DT Inhibition	.147	.161	.090
5DT Flexibility	.202	.230	.100
L&N	−.228	−.251	−.138
Spatial L Forward	−.051	−.055	−.033
Spatial L Backwards	−.094	−.150	0.72
Digit Forward	−.435^**^	−.445^**^	−.356^*^
Digit Backwards	.020	−.010	−.099
False Pos 1-back	−.191	.181	−.188
False Pos 2-back	.200	.191	.195
% Correct 1-back	−.097	−.122	−.019
% Correct 2-back	−.233	−.251	−.155

**Notes.**

FSQ I.2 Fibromyalgia Survey Questionnaire “Trouble thinking or remembering” MFE-30 Memory Failures in Everyday Questionnaire DOT Digit self-Ordering Task 5DT 5-Digit Test L&N Letter-Number Sequencing Subtest Spatial L. Spatial Localization Subtest False Pos false positives

* *p* < .05.

** *p* < .01.

## Discussion

This study examined the objective performance of working memory (WM) tasks and the subjective cognitive complaints in a group of female FM patients. The patients were compared to a healthy control group of women matched for age, educational level and laterality, as well as menopausal status and speed of information processing. Possible effects of pain, depression and fatigue on group differences were also taken into account.

Overall, the results indicate that FM patients do not differ from healthy controls in WM functioning. Only the performance of a task related to visuospatial WM was significantly poorer in the FM patients than in controls, and the differences were mediated by the presence of depressive symptoms, fatigue and pain. Analysis of the individual performances revealed deficient execution of this visuospatial task in almost half of the FM patients. Regarding subjective cognitive complaints, and as expected, patients showed a greater perception of cognitive difficulties than healthy controls, even in activities of daily living. This difference was independent of pain but was explained by depression and fatigue. Furthermore, cognitive complaints in FM patients were only associated with a lower verbal WM capacity.

The results of our study show that FM patients did not perform less well than healthy controls in terms of the global outcome across all the WM tasks. This finding is consistent with those of earlier studies using tasks related either to the phonological loop (Landrø, Stiles & Sletvold, 1997; Suhr, 2003; Luerding et al., 2008; Walteros et al., 2011; Kim et al., 2012; Cherry et al., 2014; Coppieters et al., 2015), the visuospatial sketchpad (Kim et al., 2012), monitoring and updating information capacities (Suhr, 2003; Ceko, Bushnell & Gracely, 2012; Tesio et al., 2015), the ability to manipulate verbal information online (Landrø, Stiles & Sletvold, 1997; Roldán-Tapia et al., 2007; Luerding et al., 2008; Kim et al., 2012; Cherry et al., 2014), or inhibition and attentional flexibility capacities (Suhr, 2003; Walteros et al., 2011; Glass et al., 2011; Mohs et al., 2012; Veldhuijzen, Sondaal & Oosterman, 2012; Martinsen et al., 2014; Schmidt-Wilcke et al., 2014; Cherry et al., 2014). However, our findings contrast with those of previous studies showing impairment on WM performance in FM patients (Dick, Eccleston & Crombez, 2002; Roldán-Tapia et al., 2007; Leavitt & Katz, 2008; Munguía-Izquierdo et al., 2008; Luerding et al., 2008; Seo et al., 2012; Akdoğan et al., 2013; Martinsen et al., 2014; Coppieters et al., 2015; Di Tella et al., 2015). There are several possible explanations for this discrepancy. First, some authors of previous studies have interpreted group differences in processing speed scores as WM deficits (Veldhuijzen, Sondaal & Oosterman, 2012; Akdoğan et al., 2013; Martinsen et al., 2014; Cherry et al., 2014; Coppieters et al., 2015; Tesio et al., 2015), while we considered the accuracy scores. Second, other authors did not specify the WM domains assessed when tasks involved different scores (Suhr, 2003; Luerding et al., 2008; Walteros et al., 2011), whereas we associated each score with a WM domain. Finally, some authors compared cognitive performance of FM patients with normative data (Landrø, Stiles & Sletvold, 1997; Luerding et al., 2008), whereas we used a matched control group.

The overall performance of patients was comparable to that of controls in the tasks evaluating the above-mentioned domains; however, the patients obtained a lower span score in the *Backwards Spatial Localization Subtest* (Wechsler, 1997), which is related to the ability to maintain and manipulate visuospatial information. Although scarce attention has been paid to the visuospatial WM domain in previous research, Kim et al. (2012) obtained similar results using a computerized version of the task. Luerding et al. (2008) also reported similar findings, although they did not specify whether they used the forward or backwards part of the task; these authors also used normative data.

We observed that the significant difference between the groups in visuospatial WM span can be explained by the intensity of fatigue, depression, and pain reported by FM patients, as previously suggested (Kim et al., 2012). As these symptoms are diagnostic criteria for FM, intrinsic characteristics of the disease seem to mediate poor performance in visuospatial WM.

Analysis of the individual performance of FM patients showed that almost half of the patients showed deficient performance in the *Backwards Spatial Localization Subtest*; thus, in their capacity to manipulate visuopatial information while in temporary storage. These findings also showed that a fifth of the patients displayed deficient performance in the forward part of the test, related to the visuospatial sketchpad, and also in the correct response score of the *1-back* task. Given that a deficient performance was not observed in the false positive score in this task, it is possible that updating, but not inhibition, capacities are affected in FM patients. However, the exploratory nature of this analysis, the lack of such deficiency in the *2-back* task (maybe due to practice effects caused by the order of task administration), and the scarcity of previous data, make interpretation of the findings difficult. Only two previous studies analysed individual performance to study cognition in FM (Tesio et al., 2015; Di Tella et al., 2015) and the data are not comparable to the present data. The previous studies did not include visuospatial working memory measures and used normative data to establish individual performance.

Regarding subjective cognitive complaints, the findings also reveal a widespread presence of subjective cognitive complaints in FM patients. They differed from controls in the perception of their overall memory state, measured by the *FSQ I.2*. From a more detailed perspective, FM patients also reported more cognitive complaints, through items of the *MFE-30*, both in the Total score and in specific scores related to general functioning and daily living. These findings represent evidence of the ubiquity of the concern about cognitive functioning expressed by FM patients, as reflected in previous studies (Grace et al., 1999; Glass & Park, 2001; Park et al., 2001; Glass et al., 2005; Castel et al., 2008; Arnold et al., 2008; Williams, Clauw & Glass, 2011; Tesio et al., 2015; Kravitz & Katz, 2015; Walitt et al., 2016; Gelonch et al., 2016; Schmaling & Betterton, 2016).

Our results also showed that the differences in cognitive complaints between FM patients and controls are explained by fatigue and depressive symptoms, but not by pain. The influence of mood symptoms and fatigue in subjective perception of cognitive functioning has also been reported in other studies with FM patients and other clinical populations (Castel et al., 2008; Williams, Clauw & Glass, 2011; Svendsen et al., 2012; Balash et al., 2013; Chin et al., 2014; Walitt et al., 2016; Gelonch et al., 2016; Gelonch et al., 2017). Studies have shown that memory complaints in patients with chronic pain are not related to pain intensity, but have been related to mood conflicts, and specially depression (Jamison, Sbrocco & Parris, 1989; McCracken & Iverson, 2001). Therefore, our results seem to agree that the perceived cognitive impairment does not appear to be a consequence of pain but is part of a cluster of symptoms related to fatigue and mood disorders present in many chronic diseases, including fibromyalgia. This symptomatology may exacerbate the feeling in these patients that they are not capable, or that they require a greater effort in the short term to perform a certain cognitive task (Bar-On Kalfon et al., 2016). This should be considered when developing intervention strategies in FM patients, since treatments aimed at reducing physical and affective symptoms could also lead to improvements in their subjective cognitive perception.

According to data from previous studies (Walitt et al., 2016; Gelonch et al., 2016), our results show a discrepancy between the scarce objective WM deficits and the broad presence of subjective cognitive impairment in FM patients. This discrepancy is consistent with the idea that subjective and objective measures encompass different neural processes (Gelonch et al., 2017), as suggested in studies showing altered brain activity in FM patients when performing WM tasks, even in the absence of behavioural impairment (Luerding et al., 2008; Glass et al., 2011; Ceko, Bushnell & Gracely, 2012; Seo et al., 2012; Schmidt-Wilcke et al., 2014; Walitt et al., 2016; González-Villar et al., 2017).

Results of the correlational analyses between measures of WM objective performance and cognitive complaints also support the discrepancy between the objective and subjective outcomes in FM patients. They only showed negative relationships between the *Digit Forward Subtest* span and the *MFE-30* scores. Thus, a lower capacity of verbal WM may be related to more cognitive complaints in FM patients, as also observed in a previous study (Park et al., 2001).

One possible explanation for the marked differences between the objective and subjective outcomes in FM patients is that the patients may develop compensatory strategies (Jessen et al., 2014) and thus overcome the cognitive impairment during a single task session, as a one-off effort. However, the patients cannot sustain this level of exertion in their daily lives, and therefore they report day-to-day difficulties (Williams, Clauw & Glass, 2011). Overexertion may also explain other symptoms characteristic of the disease, such as the high levels of fatigue and depression, which we found to explain the cognitive complaints in the FM patients in the present study. Further research is needed to clarify this hypothesis and the role of characteristic symptoms of the disease on both objective and subjective cognitive measures in FM patients.

One limitation to consider in interpreting our results is that, as in many studies involving patients with chronic pain, we were faced with the difficult challenge of monitoring medication intake. These patients do not tend to be constant in their intake, are polymedicated, and their prescribed drugs are continuously being modified due to the slight or lack of efficacy of their medication. Participants were asked not to take more drugs than necessary but, for ethical reasons, the prescribed medication was not withdrawn. While this lack of control may have effects—positive or negative—on cognitive functioning, temporary discontinuation of medication may also induce negative effects on cognitive function or alterations in brain activity. Nonetheless, the study sample was representative of FM patients, who very often take combinations of drugs. Another limitation of this study is that we count on a modest sized sample, although groups were well matched. This limitation is present in large part of the literature concerning cognitive dysfunction in fibromyalgia and stresses the need to conduct studies with larger samples in the future.

Finally, from a clinical perspective, the findings of our work contribute to the accumulated evidence that there is a need to increase the interest in FM dyscognition. Future research approaches should consider the importance of cognitive complaints and the, although punctual, cognitive impairment in FM patients. It would be worthwhile to characterize these patients through studies with larger samples, including a complete neuropsychological assessment and a functional impact evaluation of both their objective and subjective cognitive status. With this approach, research could ultimately study in FM patients the presence of Minor Neurocognitive Disorder (American Psychiatric Association, 2013).

## Conclusion

This study highlights how difficult it is to show a large objective alteration in WM performance, apart from an occasional deficit in a specific component, how easy it is to show a subjective difference, and how poorly these are correlated in patients with fibromyalgia.

##  Supplemental Information

10.7717/peerj.5907/supp-1 Supplemental Information 1 Working memory performance, subjective cognitive complaints and variables in fibromyalgia patients

## Additional Information and Declarations

### Data Availability

The following information was supplied regarding data availability: The raw data are available in the Supplemental Information 1.
